# Progression of the Immune Escape Mechanism in Tumors

**DOI:** 10.3390/biology13110898

**Published:** 2024-11-04

**Authors:** Sheila Spada, Sumit Mukherjee

**Affiliations:** 1 Tumor Immunology and Immunotherapy Unit, IRCCS-Regina Elena National Cancer Institute, 00144 Rome, Italy; 2 Department of Cardiothoracic and Vascular Surgery, Albert Einstein College of Medicine, Bronx, NY 10461, USA

There exists a long-standing research interest to understand the molecular and signaling interactions between tumor cells and the innate and adaptive immune cells such as dendritic cells, macrophages, NK cells, and B and T cells that occur in the tumor microenvironment (TME) [1]. The onset of immunosuppression via various mechanisms in these immune cells in the TME can potentially result in cancer cells escaping the antitumor immune responses emanating from these immune cells. The phenomenon of cancer immune escape results in further cancer proliferation, dissemination, and metastasis. Therefore, understanding these immune-inhibitory interactions and suppressing them can potentially trigger a robust anti-tumor immune response. Recently, understanding these immunosuppressive pathways in tumors and therapeutically targeting them has taken priority, and the inhibition of “immune checkpoint pathways” such as CTLA4, PD1, and PDL1 has revolutionized translational cancer research in patients [2].

The research topic “Progression of the Immune Escape Mechanism in Tumors” comprises five review articles.

Dutta, Ganguly, and guest editors have provided a comprehensive overview of studies conducted to identify new ICIs and have highlighted combination strategies to improve the efficacy of the widely approved ICIs. We first discussed the well-known immune checkpoints, such as PD-L1, PD-1, and CTLA-4, and then highlighted newer emerging ICIs. For example, one such newer emerging ICI is HLA-E, which is overexpressed by cancer cells and functions in immune suppression by binding CD94/NKG2A on NK and T cells. Furthermore, several clinical trials regarding the development of blocking antibodies targeting the adenosine A2A and A2B receptors, CD47-SIRPα pathway, TIM-3, LAG-3, TIGIT, and VISTA, along with newer targets such as PARPs, mARTs, and B7-H3, have been discussed, all of which contribute to immunosuppression and immunoresistance. Additionally, we have also included miRNAs, mRNA, and CRISPR-Cas9-mediated technologies such as CAR-T therapy to showcase newer immunotherapeutic approaches to identify and target cancers in patients [2].

In parallel, Mitra and colleagues analyzed cancer mechanisms of immune evasion, explored the current landscape of the developments in cancer immunology and immunotherapy, and discussed different immunotherapeutic approaches, such as cancer vaccines, adoptive cell therapy, and antibody-based treatments that harness the immune arsenal to overcome immune evasion [3].

Yu and co-authors focused on the immune escape in glioblastoma (GBM), a highly aggressive and incurable brain tumor. They elucidated the mechanisms that compromise the efficacy of ICIs and CAR/T cell therapy and reported promising preclinical data on emerging agents, such as small-molecule toosendanin and nanostructures such as nano-reshapers in combination with ICIs. They also showed that targeting phenomena such as ferroptosis and phosphoglycerate dehydrogenase (PHGDH)-mediated endothelial cell metabolism in combination with existing immunotherapy strategies can be considered for GBM patients.

Sheta et al. reviewed the crucial roles of extracellular vesicles (EVs) in immunosuppression, immune evasion, apoptosis of immune cells, and immunotherapy resistance. They also reviewed other roles of EVs in the delivery of bioactive molecules and meditating intercellular communication between cancer cells and various tumor-associated cells such as cancer-associated fibroblasts, adipocytes, blood and lymphatic vessels, and immune cells. Thus, it was shown that EVs shape cancer and non-malignant cells within the TME by generating an immunosuppressive and therapy-resistant TME. 

Mastrogiovanni and colleagues, in their review, discuss the mechanisms by which tumors trigger immune escape by altering the manners and features of immune cell adhesion. Such mechanisms include the tumor-mediated inhibition of immune cell infiltration within the TME by disabling vasculature, adhesive molecule expression, and chemokine signaling profiles. In this review, the authors also highlight the significance of inhibiting these alterations in TME immune cell adhesion by discussing clinically relevant effective drug treatments and cellular immunotherapies.

In this Special Issue, we and other groups have showcased scientific studies concerning immune escape mechanisms in cancers, with the hope that it will lead to further progress in the research field of tumor immunology and immunotherapy. We hope that this Special Issue has addressed the urgent need to study and understand novel and existing immunosuppressive pathways that can have therapeutic implications in different tumor types. We anticipate that this Special Issue will also pave the path for future studies that will utilize the gained knowledge in designing effective therapeutic tools that will ultimately help in cancer management for patients.
